# GM-CSF induces noninflammatory proliferation of microglia and disturbs electrical neuronal network rhythms in situ

**DOI:** 10.1186/s12974-020-01903-4

**Published:** 2020-08-11

**Authors:** Hasan Onur Dikmen, Marc Hemmerich, Andrea Lewen, Jan-Oliver Hollnagel, Bruno Chausse, Oliver Kann

**Affiliations:** 1 Institute of Physiology and Pathophysiology, University of Heidelberg, Im Neuenheimer Feld 326, 69120 Heidelberg, Germany; 2 Interdisciplinary Center for Neurosciences (IZN), University of Heidelberg, Heidelberg, Germany

**Keywords:** Cytokines, Electrophysiology, GM-CSF, Hippocampus, Innate immunity, Microglia, Neuroinflammation, Neuronal activity, Slice culture, Neurotransmission

## Abstract

**Background:**

The granulocyte-macrophage colony-stimulating factor (GM-CSF) (or CSF-2) is involved in myeloid cell growth and differentiation, and, possibly, a major mediator of inflammation in body tissues. The role of GM-CSF in the activation of microglia (CNS resident macrophages) and the consequent impacts on neuronal survival, excitability, and synaptic transmission are widely unknown, however. Here, we focused on electrical neuronal network rhythms in the gamma frequency band (30–70 Hz). Gamma oscillations are fundamental to higher brain functions, such as perception, attention, and memory, and they are exquisitely sensitive to metabolic and oxidative stress.

**Methods:**

We explored the effects of chronic GM-CSF exposure (72 h) on microglia in male rat organotypic hippocampal slice cultures (in situ), i.e., postnatal cortex tissue lacking leukocyte invasion (adaptive immunity). We applied extracellular electrophysiological recordings of local field potential, immunohistochemistry, design-based stereology, biochemical analysis, and pharmacological ablation of microglia.

**Results:**

GM-CSF triggered substantial proliferation of microglia (microgliosis). By contrast, the release of proinflammatory cytokines (IL-6, TNF-α) and nitric oxide, the hippocampal cytoarchitecture as well as the morphology of parvalbumin-positive inhibitory interneurons were unaffected. Notably, GM-CSF induced concentration-dependent, long-lasting disturbances of gamma oscillations, such as slowing (beta frequency band) and neural burst firing (hyperexcitability), which were not mimicked by the T lymphocyte cytokine IL-17. These disturbances were attenuated by depletion of the microglial cell population with liposome-encapsulated clodronate. In contrast to priming with the cytokine IFN-γ (type II interferon), GM-CSF did not cause inflammatory neurodegeneration when paired with the TLR4 ligand LPS.

**Conclusions:**

GM-CSF has a unique role in the activation of microglia, including the potential to induce neuronal network dysfunction. These immunomodulatory properties might contribute to cognitive impairment and/or epileptic seizure development in disease featuring elevated GM-CSF levels, blood-brain barrier leakage, and/or T cell infiltration.

## Background

Microglia are the tissue-resident macrophages of the central nervous system (CNS) that become activated in most brain disorders, such as stroke, bacterial meningoencephalitis, multiple sclerosis, and Alzheimer’s disease [1, 2]. The activation of microglia under such pathophysiological conditions is complex and associates with morphological changes, proliferation, antigen presentation, release of cytokines and free radicals, migration, and phagocytosis; the functional consequences range from neuroprotective to neurotoxic [3–5].

To sense danger signals and/or homeostatic imbalance within the brain, microglia express a variety of receptors that recognize, for example, bacterial and viral components, modified endogenous ligands, neurotransmitters, and neuromodulators [3, 6]. In addition, microglia express receptors for cytokines, chemokines, and growth factors that permit control over the complex process of microglial activation by other cell types, such as invading peripheral immune cells [6, 7].

The granulocyte-macrophage colony-stimulating factor (GM-CSF) (or CSF-2) is a member of the colony-stimulating factor superfamily involved in mammalian myelopoiesis, i.e., the generation of monocytes, macrophages, dendritic cells, and polymorphonuclear phagocytes [8]. The GM-CSF receptor triggers the activation of various intracellular signaling pathways, including JAK/STAT, MAPK, PI3K, and canonical NF-kappa B [9, 10]. Recent evidence suggests that GM-CSF is also involved in the activation of immune cells and, possibly, a major mediator of inflammatory processes in body tissues, including the CNS [7, 8, 11, 12].

Microglia express the GM-CSF receptor [13–15]. Its activation induces microglial proliferation, migration, and upregulation of surface markers, such as CD11b, which has been described in vitro and in vivo [9, 13–19]. However, the role of GM-CSF in the activation of microglia in the presence of other glial cells and functional neuronal networks, including the impact on neuronal survival, excitability, and synaptic transmission, is almost unknown [20, 21].

We addressed this basic issue in postnatal cortex tissue (in situ), i.e., organotypic hippocampal slice cultures that have been progressively used to study different phenotypes of microglia and microglia-neuron interactions [20, 22, 23]. Slice cultures feature well-preserved cytoarchitecture and functional neuronal networks, whereas they inherently lack infiltration of leukocytes from blood vessels during experimental exposures [22–24].

Electrical neuronal network rhythms in the gamma frequency band (30–70 Hz) naturally occur during cognition and behavior in various brain regions in vivo [25, 26]. We used such gamma oscillations as a sensitive functional readout of precise synaptic transmission between excitatory pyramidal neurons and inhibitory interneurons in situ [27–29].

## Methods

### Animals

Wistar rats were purchased from Charles-River (Sulzfeld, Germany) and handled in accordance with the European directive 2010/63/EU and with the consent of the animal welfare officers at the University of Heidelberg (licenses, T96/15 and T45/18). Experiments were performed and reported in accordance with the ARRIVE guidelines.

### Preparation and exposures of slice cultures

Organotypic hippocampal slice cultures were prepared as previously described [23, 30]. In brief, hippocampal slices (400 μm) were cut from the brains of male rats at postnatal day nine or ten (p9–p10) using a McIlwain tissue chopper (Mickle Laboratory Engineering Company Ltd., Guildford, UK) and under sterile conditions. Three to four slices with intact hippocampal structures were maintained on Biopore™ membranes (Millicell standing inserts, Merck Millipore, Darmstadt, Germany) at the interface between serum-containing culture medium and humidified normal atmosphere enriched with 5% CO_2_ (36.5 °C) in an incubator (Heracell, Thermoscientific, Dreieich, Germany). The culture medium consisted of 50% minimal essential medium, 25% Hank’s balanced salt solution (Sigma-Aldrich, Taufkirchen, Germany), 25% heat-inactivated horse serum (Life Technologies, Darmstadt, Germany), and 2 mM l-glutamine (Life Technologies) at pH 7.3 titrated with Trisbase. The culture medium (1 mL) was replaced three times per week.

From each preparation, membranes with slice cultures were randomly assigned to experimental groups. Slice cultures were used for electrophysiological recordings or fixed for immunohistochemistry and toluidine staining. For biochemical analysis, the “conditioned” culture medium was collected after 72 h and stored at − 80 °C.

To deplete the microglial cell population, liposome-encapsulated clodronate (Liposoma B.V., Amsterdam, The Netherlands) was continuously present in the culture medium at a final concentration of 100 μg/mL from day in vitro (DIV) 0 on [23, 29, 31].

Slice cultures were stimulated by exposures to GM-CSF, the proinflammatory cytokines interferon-gamma (IFN-γ) and interleukin 17 (IL-17), or to bacterial lipopolysaccharide (LPS). Notably, the paired exposure to IFN-γ plus LPS results in severe neuronal dysfunction and death in slice cultures and served as a positive control. The stock solutions of GM-CSF, IFN-γ, and IL-17 were prepared in sterile sodium phosphate buffer and/or culture medium. LPS (from *Escherichia coli*, serotype R515 (Re)) was ready-to-use. Aliquots of solutions were kept at − 20 °C. GM-CSF, IFN-γ, and IL-17 were purchased from PeproTech GmbH (Hamburg, Germany); LPS was from Alexis Biochemicals (Enzo Life Sciences GmbH, Lörrach, Germany). The working concentrations in different exposures are described in the results and figure legends. Note that these values refer to the respective concentration in the culture medium and that the final concentration in the tissue might be considerably lower due to diffusion dynamics and/or degradation by proteases.

### Biochemical analysis of the culture medium

All enzyme-linked immunosorbent assay (ELISA) kits were purchased from R&D (R&D Systems, Inc., Minneapolis, MN, USA) and applied according to the supplier’s protocol for the detection of interleukin 6 (IL-6; Cat. num. DY506) and tumor necrosis factor-alpha (TNF-α; Cat. num. 510). Concentrations of antibodies strictly followed the supplier’s protocol. Wash buffer consisted of 0.05% Tween 20 (Merck Millipore, Darmstadt, Germany) in phosphate-buffered saline (PBS). Capture antibodies were diluted in PBS (pH 7.2–7.4), and the reaction plate was coated overnight. The detection antibody for TNF-α was diluted in the reagent diluent, consisting of 1% bovine serum albumin in PBS (pH 7.2–7.4); the detection antibody for IL-6 was diluted in 2% normal goat serum in reagent diluent. Ten-point standard curves were constructed from nine sequential two-fold dilution steps of recombinant IL-6 (8000 pg/mL) or TNF-α (4000 pg/mL), and a negative control containing only reagent diluent. Samples were incubated in the coated reaction plate for 2 h. The detection antibody was then applied for 2 h and visualized with tetramethylbenzidine substrate solution (Moss Inc., Pasadena, USA). The development reaction was stopped with sulfuric acid, and the optical density was determined with a microplate reader (iMark Microplate Absorbance Reader, Bio-Rad Laboratories GmbH, Munich, Germany) at 450 nm (with 540 nm reference). The concentrations of TNF-α and IL-6 (pg/mL) were estimated by using the quadratic fit.

Nitric oxide (NO) release was quantified by determining the levels of the stable metabolite nitrite using a Griess reaction-based assay that was carried out with undiluted culture medium. Nine-point standard curves were constructed by two-fold dilution steps of an 80 μΜ sodium nitrite high standard (Merck Chemicals, Darmstadt, Germany). After the addition of the Griess reagent mixture (0.05% 1-naphthylethylenediamine hydrochloride, 0.5% sulfanilamide and 2.5% orthophosphoric acid), the optical density was measured in a microplate reader at 540 nm (Bio-Rad). The molarity of NO (μM) was calculated from the standard curve using a linear fit.

### Immunohistochemistry and toluidine blue staining of slice cultures

Slice cultures were fixed for at least 2 h with 4% paraformaldehyde in 0.1 M PBS (pH 6.8), incubated for 2–3 h in 30% sucrose (AppliChem GmbH, Darmstadt, Germany) and cut into 25-μm sections with a cryostat (CM1850; Leica Biosystems, Nussloch, Germany). Immunohistochemistry was conducted in free-floating sections. All primary antibodies were diluted in PBS + 0.3% Triton™ X-100 + 10% normal goat serum (Life Technologies). Secondary antibodies were diluted in 0.2% bovine serum albumin dissolved in PBS + 0.3% Triton™ X-100. Several washing steps with PBS were conducted, e.g., after blocking of unspecific binding sites or antibody applications.

For immunohistochemistry of ionized calcium-binding adapter molecule 1 (Iba1) and parvalbumin-positive interneurons (PV), unspecific immunoglobulin reactions were blocked for 1 h with 10% normal serum. Primary antibodies were rabbit polyclonal anti-Iba1 (Fujifilm-WAKO Chemicals Europe GmbH, Neuss, Germany) and mouse anti-PV (Sigma-Aldrich), all diluted 1:1000. Cryosections were exposed overnight to the primary antibody. Unspecific binding sites of the secondary antibodies were blocked for 1 h in 0.2% bovine serum albumin (Carl Roth GmbH & Co. KG, Karlsruhe, Germany). Secondary antibodies used were as follows: for Iba1, biotin-conjugated goat anti-rabbit (Vector Laboratories Inc., CA, USA) diluted 1:1000, and for PV, biotin-conjugated horse anti-mouse (Vector Laboratories) diluted 1:1000. The secondary antibody was applied overnight at 4 °C under light-protected conditions. Afterwards, sections were incubated for 2 h with 0.5% avidin and biotinylated horseradish peroxidase (Vectastain Elite ABC Kit, Vector Laboratories). Antibody binding was visualized by adding 0.05% diaminobenzidine substrate, 0.3% ammonium nickel sulfate in 0.05 M Trisbase 7-9®, and 0.003% H_2_O_2_ for < 5 min. Then, the reaction was stopped by adding PBS (when the brown color was intense enough). Stained sections were placed on object plates and dried. Sections were then exposed to ascending ethanol series, for 10 min in xylene (Sigma-Aldrich), and finally embedded with Entellan®Neu (Merck Millipore, Schwalbach, Germany).

For toluidine blue staining (Sigma-Aldrich), sections were mounted on slides, dried and exposed to descending ethanol series, briefly rinsed in double-distilled water, and then incubated in 0.1% toluidine blue working solution (pH 2.3) for 1–3 min. Thereafter, the sections were briefly rinsed in double-distilled water. Ninety-five percent ethanol with traces of glacial acetic acid was used for color differentiation of the staining. Sections were then exposed to 100% ethanol, followed by a 1:1 mixture of 100% ethanol and xylene and finally xylene for 3–10 min. Sections were embedded with Entellan®Neu (Merck Millipore, Schwalbach, Germany).

### Stereological counting of microglia

The numbers of microglia (Iba1-positive cell somas) were estimated with design-based stereology recently described for hippocampal slice cultures in detail [23]. In brief, we implemented the optical fractionator probe using the Stereo Investigator 5.65 software (MicroBrightField Europe, Delft, The Netherlands), which provides an estimator of the total particle number in a three-dimensional structure. Sequential sections (total of 4–7) of each slice culture were included in the analysis. To satisfy the coefficient of sampling error (CE) < 0.1, the optimal size of the frame-associated area and grid spacing were chosen [23]. The estimated microglia number of each slice culture, $\hat{N\ }$, was determined using the optical fractionator equation, i.e., $\hat{N} = \frac{Q}{hsf\ast asf\ast ssf}$. *Q* was the number of the counted cells in the fractionator frame associated area of all sections, *hsf* the height sampling fraction ($\frac{fractionator\ height}{section\ thickness}$), *asf* the area sampling fraction ($\frac{frame\ associated\ area}{contour\ area\ of\  all\  sections}$), and *ssf* the section sampling fraction, i.e., the interval of sections sampled through an object of interest. As we sampled every section of each slice culture, the section sampling fraction was always 1. As a result of inevitable tissue shrinkage during the staining procedures, the initial section thickness before staining (25 μm, obtained by cutting with a cryostat) had to be adjusted.

### Recording solutions and drugs

Slice cultures were constantly supplied with pre-warmed recording solution (artificial cerebrospinal fluid; ACSF). ACSF contained 129 mM NaCl, 3 mM KCl, 1.25 mM NaH_2_PO_4_, 1.8 mM MgSO_4_, 1.6 mM CaCl_2_, 21 mM NaHCO_3_, and 10 mM glucose [23, 24]. The pH was 7.3 when the recording solution was saturated with 95% O_2_ and 5% CO_2_. Recordings were done at 34 ± 1 °C.

Cholinergic gamma oscillations were elicited by continuous application of acetylcholine (2 μM) and the acetylcholine-esterase inhibitor physostigmine (400 nM) via the recording solution [24, 29]. Acetylcholine was purchased from Sigma-Aldrich; physostigmine was obtained from Tocris (R&D Systems GmbH, Wiesbaden-Nordenstadt, Germany).

### Recordings of local field potential

For electrophysiological recordings, the intact Biopore™ membrane carrying slice cultures was inserted into the recording chamber [23, 24]. Slice cultures were maintained at the interface between the recording solution and the ambient gas mixture. Intact Biopore™ membrane inserts ensure rapid and efficient supply of oxygen, energy substrates, and drugs through the recording solution (rate 1.8 mL/min) that flows underneath. The interface condition permits constant oxygen supply from the ambient gas mixture (95% O_2_ and 5% CO_2_, rate 1.5L/min). Recordings of local field potentials started during the induction phase of gamma oscillations that lasts for about 25 min in the presence of acetylcholine and physostigmine. The properties of persistent gamma oscillations (see below) and the other patterns of network activity were analyzed in data segments of 5 min recorded after the induction phase (> 30 min).

Local field potentials were recorded with glass electrodes (resistance of 1–2 MOhm) that were made from GB150F-8P borosilicate filaments (Science Products GmbH, Hofheim, Germany) using a Zeitz DMZ Puller (Zeitz-Instruments Vertriebs GmbH, Martinsried, Germany), and filled with ACSF. The electrode was positioned in the stratum pyramidale of the CA3 region with a mechanical micromanipulator (MM 33, Märzhäuser, Wetzlar, Germany). Local field potentials were recorded with an EXT 10-2F amplifier in EPMS-07 housing (npi electronic GmbH, Tamm, Germany), low-pass filtered at 3 kHz, and digitized at 10 kHz using CED 1401 interface and Spike2 software (Cambridge Electronic Design, Cambridge, UK).

### Data analysis and statistics

Offline analysis was performed in MatLab 2018b (The MathWorks, Inc., Natick, MA, USA). For gamma oscillations, data segments of 5 min were subdivided into segments of 30 s, band-pass filtered (FFT filter, pass-band frequency: 5–200 Hz) and processed with Welch’s algorithm and a fast Fourier transformation (FFT size 8192). The resulting plots of the power spectral density had a resolution of 1.2207 Hz. For calculation of the time constant, autocorrelations of data segments were fitted with an exponential decay function. Gamma oscillations were analyzed for various parameters, i.e., peak frequency (frequency), peak power (power), full width at half-maximum, and time constant. Spectrograms (time-frequency plots) were derived from continuous wavelet transforms of a given local field potential recording using Morlet wavelets. This method provides an instant measure of the power of various frequencies at any given time point, thereby offering visualization of fast dynamics in frequency and power.

Data were derived from “n” slices (slice cultures) and “N” preparations of rat pups. Statistical significance (*P* < 0.05) was determined in GraphPad Prism 8.0 (GraphPad Software, Inc., La Jolla, CA, USA). Data distribution was tested for normality with the Shapiro-Wilk test. Statistical tests are specified in the figure legends. Figures were created with MatLab, GraphPad Prism, and CorelDraw (Corel, Ottawa, Ontario, Canada).

## Results

### GM-CSF induces noninflammatory proliferation of microglia in situ

**Fig. 1:**
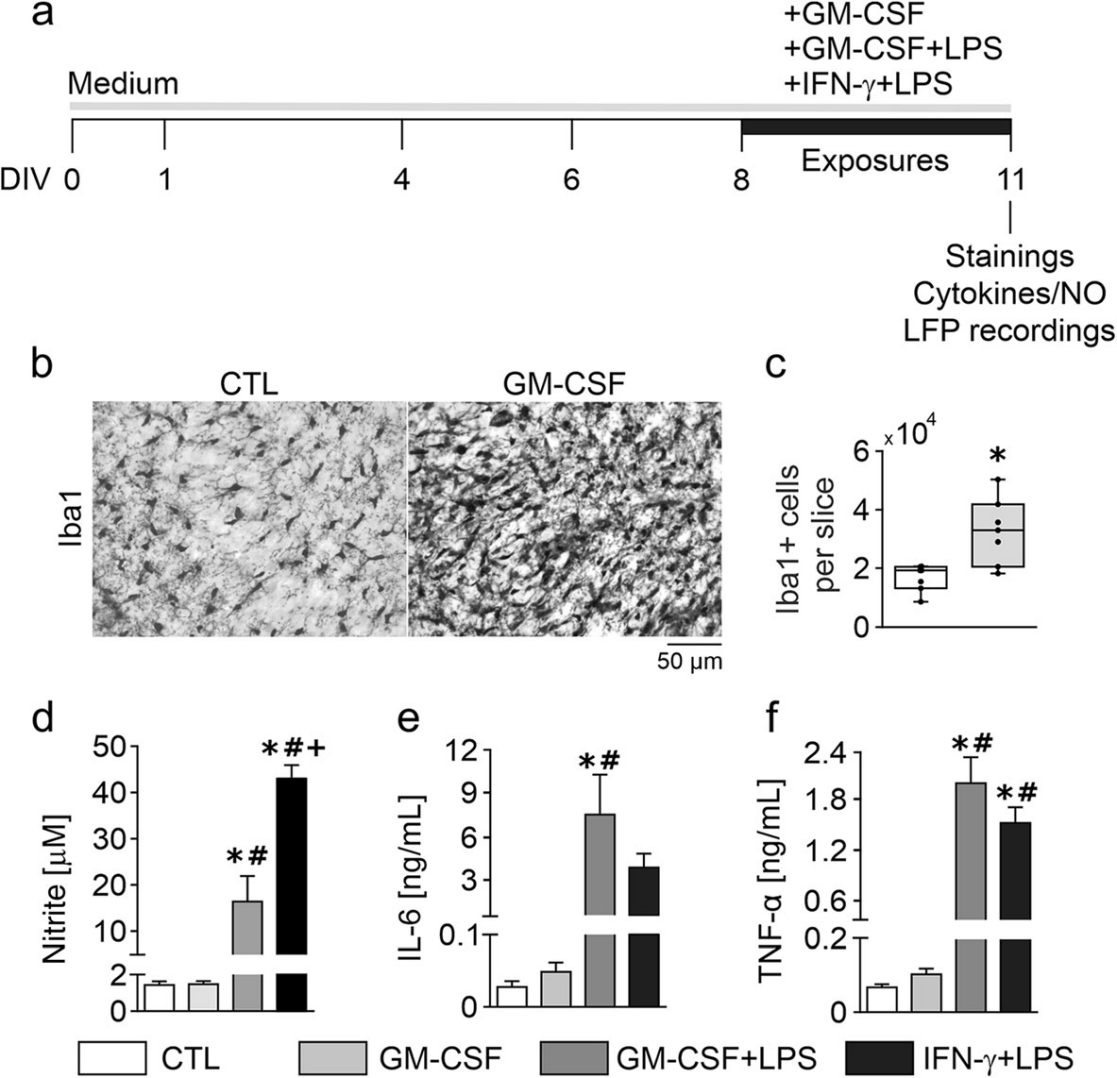
GM-CSF induces proliferation of noninflammatory microglia. **a** Experimental schedule. Slice cultures were chronically exposed to GM-CSF (100 ng/mL), GM-CSF+LPS (100 ng/mL, both) and IFN-γ+LPS (100 ng/mL, both) for 72 h from DIV 8 to DIV 11. **b** Iba1 immunoreactivity in naïve control (CTL) and GM-CSF slices. Images were taken from the hippocampal CA3 region (see scheme in Fig. 2c). **c** Quantification of Iba1-positive cells. Microglia counting was performed using a stereology-based method. **d** Nitrite accumulation in culture supernatants. **e** IL-6 and **f** TNF-α release. Note the difference in NO release between GM-CSF+LPS and IFN-γ+LPS (**d**). For **c**, values represent medians and interquartile range and the whiskers indicate minimum and maximum of data and were compared using unpaired *t*-tests. For **d**–**f**, values represent averages ± SEM and were compared using one-way ANOVA followed by Tukey’s post-test *P < 0.05 vs. CTL; #*P* < 0.05 vs. GM-CSF; +*P* < 0.05 vs. GM-CSF+LPS. For *n/N* cultures/preparations: **b** CTL, 31/8; GM-CSF, 27/7. **c** CTL, 7/2; GM-CSF, 7/2. **d** CTL, 8/5; GM-CSF, 10/5; GM-CSF+LPS, 6/3; IFN-γ+LPS, 4/3. **e** CTL, 4/4; GM-CSF, 10/4; GM-CSF+LPS, 6/3; IFN-γ+LPS, 3/3. **f** CTL, 6/4; GM-CSF, 10/5; GM-CSF+LPS, 6/3; IFN-γ+LPS, 4/3

Organotypic hippocampal slice cultures of the rat were maintained for 7 days in the incubator and then exposed for 72 h (“chronic”) to the cytokines GM-CSF and IFN-γ (type II interferon) and to bacterial LPS that served as a secondary inflammatory stimulus (Fig. 1a) [23, 29]. Thereafter, the slice cultures were either fixed for histology, including stereological analysis, or transferred to the interface recording chamber for extracellular electrophysiological local field potential recordings [23, 32]. The “conditioned” culture medium was used for biochemical analysis.

Microglia stained with the marker Iba1 showed small somata (cell bodies) and ramified morphology, including minimal territorial overlap in control slice cultures (Fig. 1b). Based on stereological counting of Iba1-positive cells, we estimated a mean population size of about 1.68 × 10^4^ microglial cells per slice (Fig. 1c).

Exposure to GM-CSF induced prominent changes in the morphology of microglia (Fig. 1b). Specifically, there was a denser and more intense Iba1 staining pattern that reflected microglial proliferation, including decreased ramification of cellular processes and increased spatial overlap of microglia. Similar morphological changes of microglia have been reported in vitro, in situ, and in vivo [13, 15, 19, 20]. The size of the microglial population increased by 1.95-fold (Fig. 1c). This level of microgliosis was similar as obtained with chronic exposure to IFN-γ (100 ng/mL, 72 h) [23]. In control and GM-CSF-exposed slice cultures, the levels of NO and the proinflammatory cytokines IL-6 and TNF-α, which had accumulated in the culture medium, were very low (Fig. 1d–f).

To further explore how GM-CSF affects microglial reactivity, we exposed slice cultures to GM-CSF plus LPS (GM-CSF+LPS) or to IFN-γ plus LPS (IFN-γ+LPS). Bacterial LPS acting through Toll-like receptor 4 (TLR4) and the proinflammatory cytokine IFN-γ are classical tools for priming and activation of microglia, and they induce severe neurodegeneration when co-applied in situ [23, 33, 34]. These paired immunological stimuli triggered robust but distinct patterns in the release of NO, IL-6, and TNF-α, indicating transitions to different proinflammatory microglial phenotypes (Fig. 1d–f). In particular, IFN-γ+LPS induced much higher NO release. Notably, single exposures to IFN-γ or LPS induce only mild increases in the release of NO in situ [20, 23, 29].

**Fig. 2:**
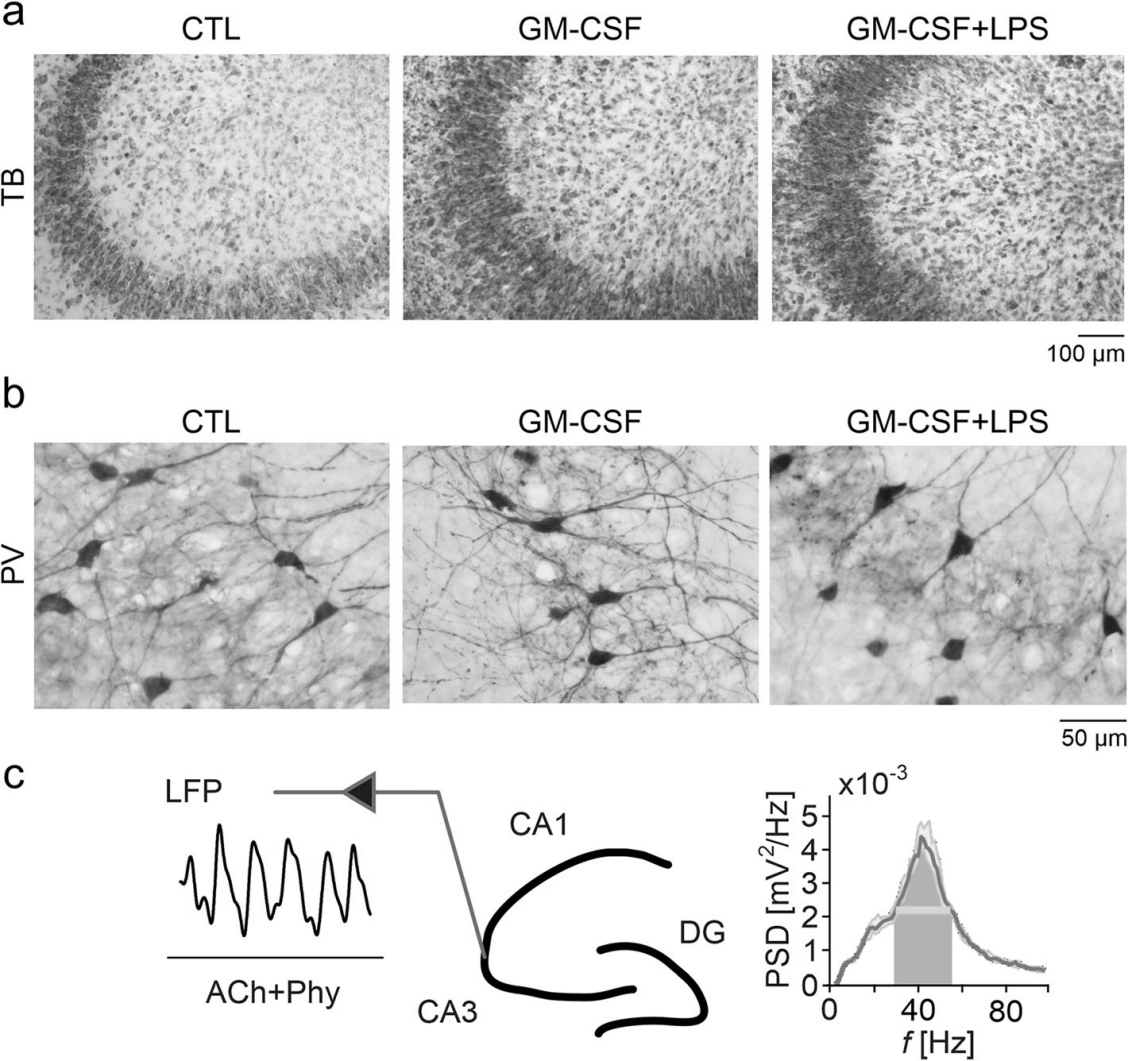
GM-CSF does not affect the hippocampal cytoarchitecture. After 72-h exposure, naïve control (CTL), GM-CSF (100 ng/mL), and GM-CSF+LPS (100 ng/mL, both) slices were stained with **a** toluidine blue (TB) for hippocampal cytoarchitecture evaluation or **b** immunostained against parvalbumin (PV) to assess inhibitory interneuron morphology. **c** Gamma oscillations were induced by acetylcholine (2 μM) and physostigmine (400 nM) (ACh+Phy) bath application at 34 ± 1 °C (*left*). Local field potential (LFP) recordings were performed in the stratum pyramidale of CA3 (*middle*) in an interface recording chamber that permits the continuous exchange of recording solution and ambient gas mixture as well as electrophysiological recordings; DG, dentate gyrus. The corresponding power spectral density (PSD) (bin size = 1.221 Hz), calculated from a data segment of 5 min, is shown as a sample (*right*). For *n/N* cultures/preparations: **a** CTL, 31/6; GM-CSF, 23/5; GM-CSF+LPS, 17/4. **b** CTL, 21/6; GM-CSF, 31/7; GM-CSF+LPS, 19/4

The cytoarchitecture of slice cultures stained with toluidine blue was well-preserved after exposures to GM-CSF or to GM-CSF+LPS. In particular, the principal cell layers, such as stratum pyramidale consisting of densely packed somata of glutamatergic pyramidal cells, were well-defined and lacked apparent malformations (Fig. 2a). Local interneurons stained for the calcium-binding protein parvalbumin were also present and contacted the perisomatic region of pyramidal cells with extensive axon arbors in control, GM-CSF and GM-CSF+LPS (Fig. 2b). Notably, functional networks of parvalbumin-positive interneurons, such as fast-spiking, GABAergic basket cells, are crucial for the generation of cholinergic neuronal gamma oscillations (see below) [28, 35]. Conversely, slice cultures exposed to IFN-γ+LPS presented severe neurodegeneration and tissue disintegration; they were not suitable for histological assessment.

These findings collectively suggest that GM-CSF induces noninflammatory proliferation of microglia (microgliosis), which also differs from priming of microglia with IFN-γ.

### GM-CSF induces disturbances of neuronal gamma oscillations in situ

To characterize the functional integrity of local neuronal networks, we recorded local field potential responses in the presence of the neurotransmitter acetylcholine (Fig. 2c) [23, 32].

**Fig. 3:**
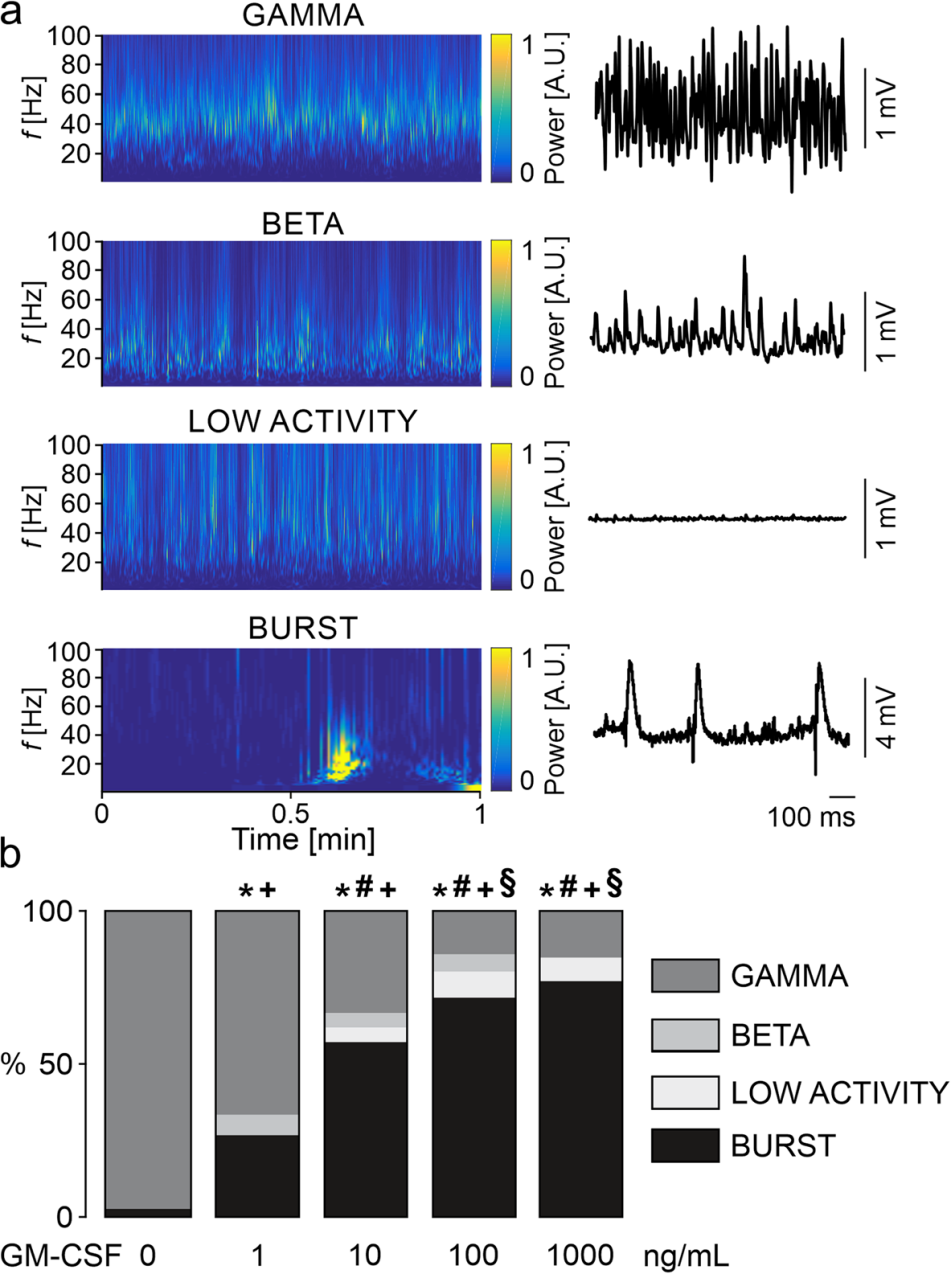
GM-CSF disturbs neuronal gamma oscillations. **a** Sample traces of the different neuronal network activities (*right*) are shown accompanied by Morlet wavelets (spectrograms, *left*) of the same state. LFP recordings were performed in the stratum pyramidale of the hippocampal CA3 region. **b** Distribution of network activities in naïve control slices (0 ng/mL) and slices exposed to different concentrations of GM-CSF. Values represent percentage of network activity type and were compared using the chi-square test. All groups were compared in respective to the presence of gamma oscillations (GAMMA vs. NO GAMMA) and recurrent neural burst firing (BURST vs. NO BURST). *P < 0.05 vs. CTL, #*P* < 0.05 vs. GM-CSF 1 ng/mL for GAMMA vs. NO GAMMA. +*P* < 0.05 vs. CTL, §*P* < 0.05 vs. GM-CSF 1 ng/mL for BURST vs. NO BURST. For *n/N* cultures/preparations: **b** CTL, 38/10; GM-CSF 1 ng/mL, 15/2; GM-CSF 10 ng/mL, 21/5; GM-CSF 100 ng/mL, 35/6; GM-CSF 1000 ng/mL, 26/6

**Fig. 4:**
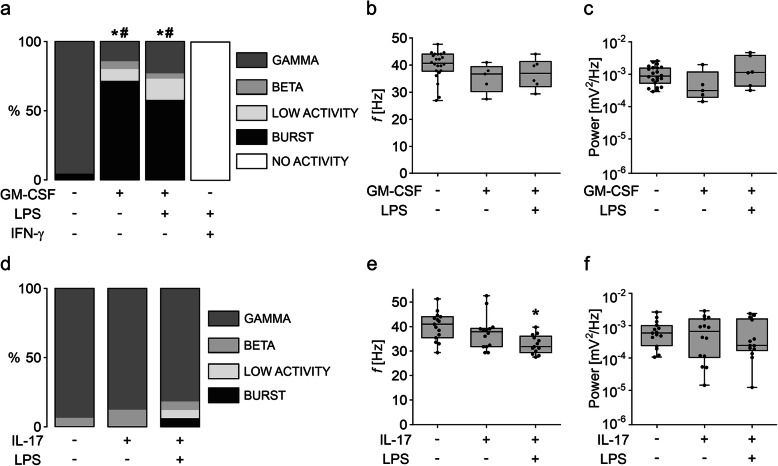
GM-CSF induces specific disturbances of gamma oscillations. **a** Distribution of network activities in naïve control slices and slices exposed to GM-CSF (100 ng/mL), GM-CSF+LPS (100 ng/mL, both), or IFN-γ+LPS (100 ng/mL, both). Gamma oscillation properties: **b** Peak frequency and **c** peak power. **d** Distribution of network activities of naïve control slices and slices exposed to IL-17 (100 ng/mL) or IL-17+LPS (100 ng/mL, both). Gamma oscillation properties: **e** Peak frequency and **f** peak power. For **a** and **d**, values represent percentage of network activity type and were compared using the chi-square test. All groups were compared in respective to the presence of gamma oscillations (GAMMA vs. NO GAMMA) and recurrent neural burst firing (BURST vs. NO BURST). All IFN-γ+LPS slices presented only “no activity” and were not included in statistical analysis. *P < 0.05 vs. CTL for GAMMA vs. NO GAMMA. #*P* < 0.05 vs. CTL for BURST vs. NO BURST. For **b**–**c** and **e**–**f**, values represent medians and interquartile range and the whiskers indicate minimum and maximum of data and were compared using one-way ANOVA with Tukey’s post hoc test. *P < 0.05 vs. CTL. For *n/N* cultures/preparations: **a** CTL, 23/6; GM-CSF, 35/6; GM-CSF+LPS, 26/3; IFN-γ+LPS, 10/3. **b**, **c** CTL, 22/6; GM-CSF, 5/2; GM-CSF+LPS, 6/3. **d** CTL, 16/3; IL-17, 16/3; IL-17+LPS, 16/3. **e**, **f** CTL, 15/3; IL-17, 14/3; IL-17+LPS, 13/3

Slice cultures usually show spontaneous asynchronous neuronal network activity in the absence of exogenous neurotransmitter receptor ligands [36, 37]. The continuous application of acetylcholine in slices enhances neural excitability and mimics cholinergic input to the hippocampus during exploratory behavior in vivo [27]. Acetylcholine reliably induced persistent gamma oscillations in control slices that had a frequency of around 40 Hz and were quite stable over time in virtually all control slice cultures tested (Figs. 3a, b and 4b). Notably, such cholinergic gamma oscillations in situ share many features with gamma oscillations in vivo and require both glutamatergic excitation and fast rhythmic GABAergic inhibition [24, 27].

Exposure to GM-CSF induced additional patterns of neuronal network activity. These patterns comprised slower oscillations in the beta frequency band (around 19 Hz), asynchronous low-voltage activity, and recurrent neural burst firing (Fig. 3a). These disturbances of neuronal network function were dependent on the concentration of GM-CSF (1 to 1000 ng/mL) (Fig. 3b). Notably, recurrent neural burst firing dominated the additional activity patterns in GM-CSF, suggesting a progressive loss of the neuronal excitation-inhibition balance in the local networks.

To further test for GM-CSF-specific effects, we explored neuronal network activity in slice cultures exposed to GM-CSF+LPS and IFN-γ+LPS. Although GM-CSF+LPS increased the release of NO, IL-6, and TNF-α (Fig. 1d–f), it did not exacerbate the disturbances of gamma oscillations (Fig. 4a). In the fraction of slice cultures that still expressed gamma oscillations, frequency and power of the oscillations were still unchanged (Fig. 4b, c). By contrast, IFN-γ+LPS resulted in complete loss of electrical activity because of severe neurodegeneration (Fig. 4a).

GM-CSF interacts with the proinflammatory cytokine IL-17 in the induction and exacerbation of neuronal disturbances in inflammatory diseases such as multiple sclerosis [38–41]. To test whether IL-17 also disturbs neuronal network activity, we exposed slice cultures to IL-17 (100 ng/mL) alone or paired with LPS (IL-17+LPS) for 72 h. Interestingly, IL-17 or IL-17+LPS had no significant effect on the incidence of gamma oscillations and only marginally affected the properties of gamma oscillations (Fig. 4d–f). Indeed, a mild decrease in the frequency was detected in IL-17+LPS, whereas the power was unchanged (Fig. 4e, f).

These foregoing findings suggest that GM-CSF induces specific disturbances of neuronal gamma oscillations dominated by recurrent neural burst firing, which are not dependent on the release of proinflammatory mediators.

### Disturbances of gamma oscillations are long-lasting and attenuated by microglia-depletion

To gain insight into the mechanisms underlying GM-CSF induced disturbances of gamma oscillations, we performed recovery and microglia-depletion experiments.

**Fig. 5:**
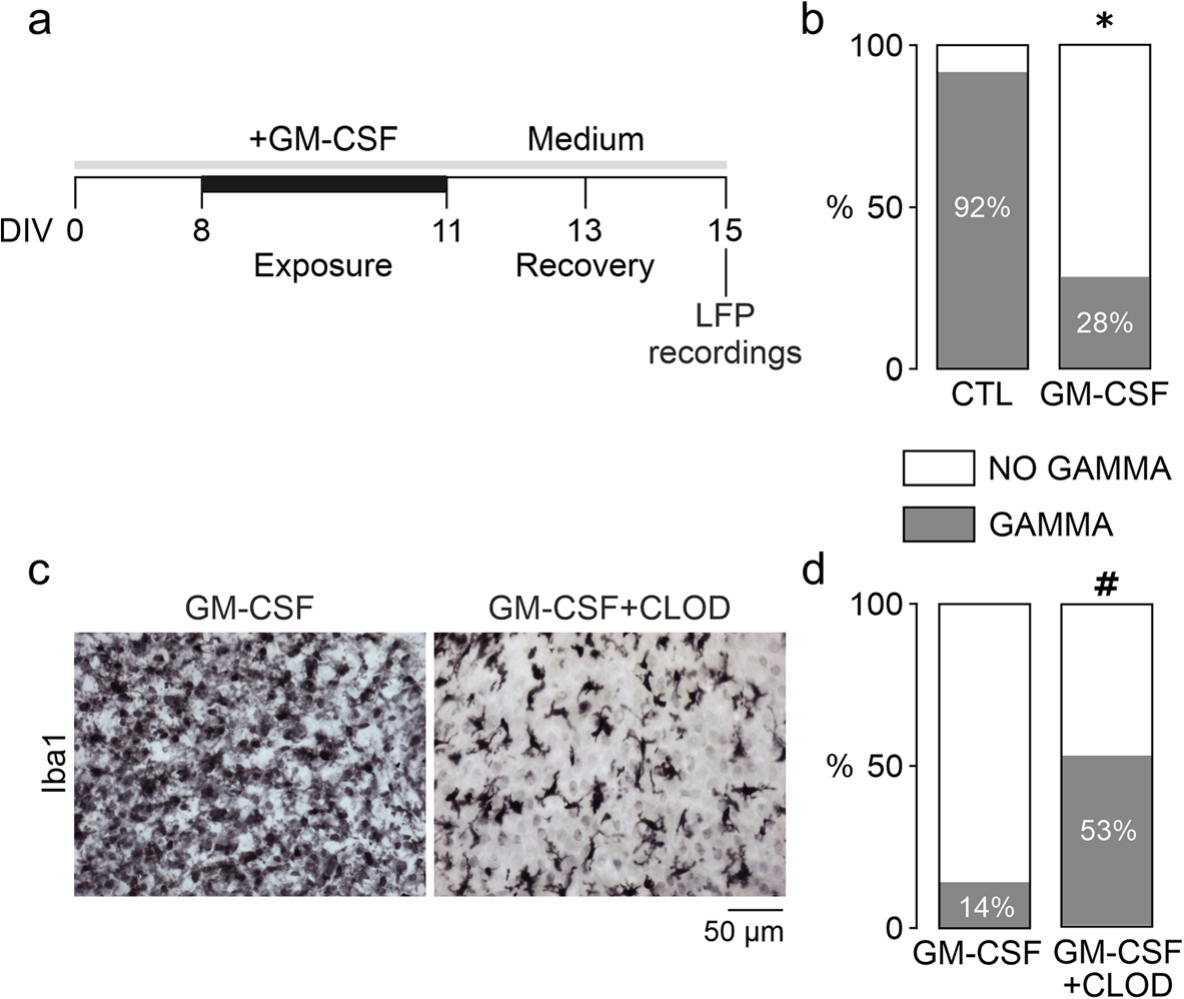
GM-CSF effects are long-lasting and attenuated by microglial depletion. **a** For the recovery experiment, slices were incubated with standard culture medium for further 96 h (from DIV 11 to DIV 15) after 72-h exposure to GM-CSF (100 ng/mL) (from DIV 8 to DIV 11) (membranes were washed once in culture medium before medium replacement). Naïve control slices were exposed to standard culture medium only. **b** Percentage of slices presenting gamma oscillations after the recovery time. For microglia-depletion experiments, slices were exposed to 100 ng/mL GM-CSF alone (GM-CSF) or in the presence of 100 μg/mL clodronate-filled liposomes (GM-CSF+CLOD). **c** Immunohistochemistry against Iba1. Sample images are taken from the CA3 region. Note that there is only a partial depletion of the microglial cell population (*right*). **d** Percentage of slices presenting gamma oscillations upon exposure to GM-CSF or GM-CSF plus clodronate. Values represent percentage of gamma oscillation appearance and were compared using the chi-square test (GAMMA vs. NO GAMMA). *P < 0.05 vs. CTL; #*P* < 0.05 vs. GM-CSF. Note that the fractions of slices expressing gamma oscillations are similar to CTL, GM-CSF (and GM-CSF+LPS) in Fig. 4a. For *n/N* cultures/preparations: **b** CTL recovery, 12/3; GM-CSF recovery, 25/3; **c** GM-CSF, 27/7; GM-CSF+CLOD, 9/3; **d** GM-CSF, 35/6; GM-CSF+CLOD, 17/3

After the maintenance for 72 h in the absence (control) or presence of GM-CSF, slice cultures were washed with culture medium and then incubated for further 96 h in standard culture medium to explore the potential recovery of neuronal network dysfunction (Fig. 5a). In this experiment, only about one-third of the slice cultures showed gamma oscillations after the recovery from GM-CSF (Fig. 5b). By contrast, the majority of control slice cultures expressed gamma oscillations, indicative for the general preservation of functional neuronal networks in situ [32, 42].

To finally assess the role of microglia, we exposed slice cultures to liposome-encapsulated clodronate [31]. This nontoxic bisphosphonate induces apoptosis specifically in macrophages and microglia after ingestion and intracellular accumulation. Clodronate was continuously present in the culture medium from DIV 0, which results in effective depletion of the microglial cell population by > 95%, without alterations of the hippocampal cytoarchitecture [23, 29]. Notably, neuronal gamma oscillations remain regular after depletion of microglia in otherwise untreated slice cultures, suggesting that non-reactive microglia are dispensable for neuronal signaling and rhythm generation in postnatal cortex tissue [43]. Exposure to GM-CSF still triggered proliferation of the residual microglia in the presence of clodronate (Fig. 5c), similar to IFN-γ [29]. However, this level of microglial depletion associated with the 3-fold increase in the fraction of slice cultures expressing gamma oscillations (Fig. 5d).

Taken together, these findings suggest that the GM-CSF-induced neuronal network dysfunction is long-lasting but substantially attenuated by microglia-depletion.

## Discussion

We explored the role of GM-CSF in postnatal cortex tissue. Our main finding is that GM-CSF triggered noninflammatory microgliosis associated with long-lasting disturbances of neuronal network rhythms.

### Exploring microglia-neuron interactions in situ

We investigated the effects of GM-CSF on microglia-neuron interactions in organotypic hippocampal slice cultures of the rat, which feature well-preserved cytoarchitecture and functional neuronal networks (Figs. 2 and 3) [30, 32, 37, 44]. Slice cultures inherently lack leukocyte infiltration during experimental exposures and thus permit to explore the reactivity of microglia in the absence of invading granulocytes, monocytes, and lymphocytes [22–24]. Importantly, microglia showed ramified morphology, minimal territorial overlap, and low release of proinflammatory cytokines in naïve control slice cultures (Fig. 1). These features reflect a widely non-reactive microglial phenotype in situ, which transformed into distinct reactive states during exposures to inflammatory mediators (Fig. 1) [22, 23, 45, 46].

In addition to featuring non-reactive microglia, slice cultures express gamma oscillations, which share many features with hippocampal acute slices and the hippocampus in vivo, such as oscillation generation in the CA3 region and average frequency of around 40 Hz (Figs. 2 and 3) [24, 27, 47, 48]. Gamma oscillations require precise chemical and electrical synaptic transmission between excitatory pyramidal cells and inhibitory interneurons [26, 27]. This neuronal network rhythm emerges in many brain areas during sensory perception, selective attention, voluntary movement, and memory formation [28, 49, 50]. Notably, gamma oscillations are exquisitely sensitive to metabolic and oxidative stress [24, 43, 51] and thus provide a sensitive functional readout of precise neuronal network signaling in situ [27–29].

We describe that GM-CSF induced severe and long-lasting disturbances in gamma oscillations, with the predominant incidence of recurrent neural burst firing. The most likely pathophysiological sequence in the development of these disturbances was slowing of the oscillations, recurrent neural burst firing, and asynchronous low-voltage activity (Figs. 3 and 4). Slowing of oscillations and particularly neural burst firing reflect alterations in the neuronal excitation-inhibition balance in local networks. It is likely that fast-spiking, parvalbumin-positive GABAergic basket cells are one of the first neuronal subtypes that were affected during exposures to GM-CSF [29, 52, 53]. Activation of microglia by GM-CSF might thus contribute to cognitive impairment [54, 55] and/or the development of noninflammatory epileptic seizures [56]. Importantly, GM-CSF and GM-CSF+LPS did not result in the absence of electrical activity (severe neurodegeneration) as typically caused by IFN-γ+LPS [23, 29].

### Effects of GM-CSF on microglia and neuronal network function in situ

GM-CSF is a monomeric glycoprotein that functions as a cytokine. In contrast to other colony-stimulating factors, GM-CSF is virtually undetectable in the systemic circulation [57]. However, growing evidence suggests that GM-CSF is produced in disease and locally active at sites of tissue inflammation, for example, in late-phase cutaneous reactions, rheumatoid arthritis, inflammatory pain, and multiple sclerosis [58–63].

GM-CSF can be released by different cell types, such as Th17 cells, GM-CSF-producer CD4+ T cells, epithelial cells, endothelial cells, fibroblasts, and, perhaps, astrocytes [7, 8, 11, 64, 65]. Notably, GM-CSF has been reported to cross the blood-brain barrier and the blood-spinal cord barrier through a saturable mechanism [66]. In the brain, GM-CSF mainly acts on microglia [13–15] and neurons [67, 68]. The presence of GM-CSF receptors in astrocytes is controversial [13, 16, 69–72].

We report that GM-CSF (100 ng/mL, 72 h) expands the microglial cell population by 1.95-fold thus having a substantial mitogenic effect in vitro and in situ [13, 15, 20]. Notably, GM-CSF induced long-lasting neuronal network dysfunction, despite failing to trigger the release of NO and proinflammatory cytokines (Fig. 1). Moreover, paired exposure to GM-CSF and LPS that served as a secondary inflammatory stimulus did not exacerbate neuronal network dysfunction, despite the increased release of NO, IL-6, and TNF-α. Interestingly, IFN-γ (100 ng/mL, 72 h) also expands the microglial cell population by 1.9-fold [29]. This priming of microglia associates only with slowing of gamma oscillations still within the gamma frequency band (30–70 Hz) and is mainly caused by moderate microglial NO release [29]. Notably, paired exposures to IFN-γ and LPS result in severe neurodegeneration in situ, primarily caused by massive microglial release of NO and reactive oxygen species [20, 23, 73]. Although GM-CSF has been recently associated with inflammation in different body tissues, our findings might differ from other studies because we investigated the responses of microglia in their natural environment with functional neuronal networks and other glial cells, but in the absence of leukocyte infiltration [23, 29, 31]. Collectively, these findings suggest that GM-CSF can exert a unique role in the activation of microglia that is distinct from the classical proinflammatory activation induced by TLR4 stimulation or priming with the T cell cytokine IFN-γ [33, 74–77].

In vivo, GM-CSF interacts with the cytokine IL-17 in the induction and exacerbation of neuronal disturbances in rodent models of multiple sclerosis [41, 78, 79]. Although the role of both cytokines on disease induction and composition of brain-infiltrating cells has been defined [11, 14, 80, 81], their effects on microglia-mediated neuronal dysfunction are still controversial [41, 82]. Here, we show that slice cultures exposed to IL-17 present regular gamma oscillations that are only mildly affected upon paired exposure with LPS (Fig. 4). This goes in line with studies in mice featuring constitutive IL-17 expression in the brain and showing microgliosis without signs of neurological dysfunction or neurodegeneration [82, 83]. By contrast, GM-CSF induced progressive disturbances in gamma oscillations, supporting the notion on the specificity of GM-CSF-mediated neuronal network dysfunction. These results suggest that GM-CSF might have a role in disease development in vivo and represent a potential target for the development of new treatments.

We also describe that the long-lasting effects induced by GM-CSF are likely mediated by microglia (Fig. 5). Notably, GM-CSF induced significant proliferation of microglia, despite the presence of clodronate. We observed a similar effect with IFN-γ, which likely reflects the high self-renewal capacity of microglia [29, 84, 85]. The functional state of the residual proliferating microglia is diverse and might differ in the presence of GM-CSF and IFN-γ [86, 87]. However, we observed a clear attenuation of neuronal network dysfunction when the microglial cell population was partially depleted. This suggests that microglia have a crucial role in GM-CSF-induced disturbances of neuronal gamma oscillations. Thus, GM-CSF might be involved in microglia-mediated neuronal dysfunction rather than tissue repair [88]. The absence of inflammation during GM-CSF exposures suggests that additional mechanisms might promote the disturbances in the gamma rhythm. These putative mechanisms include direct microglial actions on neurons, such as enhanced phagocytosis or displacement of inhibitory synapses [54, 89], which might be related to neuronal excitation/inhibition imbalance and lead to recurrent neural burst firing. Moreover, microglia might induce neuronal dysfunction by promoting neurotoxic activities in astrocytes [90, 91] and/or by altering brain energy metabolism [45, 92–94].

## Conclusion

Our data support the biological concept that GM-CSF has a unique role in the activation of microglia, including the potential to induce neuronal network dysfunction. These immunomodulatory properties might contribute to cognitive impairment and/or development of epileptic seizures in disease featuring elevated GM-CSF levels, blood-brain barrier leakage, and/or T cell infiltration.

## Data Availability

The datasets used and/or analyzed during the current study are available from the corresponding author on reasonable request.

## Abbreviations

ACh: Acetylcholine
ACSF: Artificial cerebrospinal fluid
CD11b: Cluster of differentiation molecule 11b
CLOD: Clodronate
CNS: Central nervous system
DIV: Day in vitro
GM-CSF: Granulocyte-macrophage colony-stimulating factor
Iba1: Ionized calcium-binding adapter molecule 1
IFN-γ: Interferon-gamma
IL-6: Interleukin 6
IL-17: Interleukin 17
JAK: Janus kinase
LFP: Local field potential
LPS: Lipopolysaccharide
MAPK: Mitogen-activated protein kinase
NF-kappa B: Nuclear factor kappa-light-chain-enhancer of activated B cells
NO: Nitric oxide
PBS: Phosphate-buffered saline
Phy: Physostigmine
PI3K: Phosphoinositide 3-kinase
PV: Parvalbumin
STAT: Signal transducer and activator of transcription
TLR4: Toll-like receptor 4
TNF-α: Tumor necrosis factor-alpha
